# Blood Pressure and Body Weight Have Different Effects on Pulse Wave Velocity and Cardiac Mass in Children

**DOI:** 10.3390/jcm9092954

**Published:** 2020-09-12

**Authors:** Simonetta Genovesi, Paolo Salvi, Elisa Nava, Elena Tassistro, Marco Giussani, Ilaria Desimone, Antonina Orlando, Mariagrazia Battaglino, Giulia Lieti, Massimo Montemerlo, Laura Antolini, Gianfranco Parati

**Affiliations:** 1 School of Medicine and Surgery, University of Milano-Bicocca, 20900 Monza, Italy; m.battaglino@campus.unimib.it (M.B.); g.lieti@campus.unimib.it (G.L.); gianfranco.parati@unimib.it (G.P.); 2 Istituto Auxologico Italiano, IRCCS, Cardiologic Unit, 20100 Milan, Italy; psalvi.md@gmail.com (P.S.); antonina.orlando@unimib.it (A.O.); m-montemerlo@auxologico.it (M.M.); 3 Nephrology and Dialysis Unit, IRCCS Multimedica, 20099 Sesto S. Giovanni, Italy; elisa.nava4@virgilio.it; 4 Bicocca Center of Bioinformatics, Biostatistics and Bioimaging (B4 center), School of Medicine and Surgery, University of Milano-Bicocca, 20900 Monza, Italy; e.tassistro@campus.unimib.it (E.T.); laura.antolini@unimib.it (L.A.); 5 Family Pediatrician, Agenzia Tutela Salute of Milan, 20100 Milan, Italy; abrjg@tin.it; 6 Division of Nephrology and Dialysis, Magenta Hospital, ASST Ovest Milan, 20025 Legnano, Italy; desimone.ila@gmail.com

**Keywords:** blood pressure, body mass index, children, left ventricular mass index, carotid-femoral pulse wave velocity, carotid-radial pulse wave velocity

## Abstract

Background: High blood pressure (BP) and excess weight can lead to early cardiovascular organ damage already in children. Carotid-femoral pulse wave velocity (cf-PWV) is the non-invasive gold standard method for assessing aortic stiffness, while carotid-radial PWV (cr-PWV) provides information on the distensibility of the upper limb arteries. The aim of this study was to evaluate the relationship of BP and BMI z-scores with arterial stiffness and left ventricular mass index (LVMI) in a pediatric population. Methods: In 343 children (57.7% males; age ± SD 11.7 ± 2.9 years), systolic (SBP) and diastolic (DBP) BP, BMI, cf-PWV, cr-PWV and LVMI were measured. A multiple linear regression model was used to assess the impact of BMI and SBP (or DBP) z-scores on cf-PWV, cr-PWV and LVMI. Results: About 21% of children were normal weight, 34% were overweight and 45% obese. Adjusted for possible confounders, SBP and DBP z-scores were significantly associated with cf-PWV (*p* < 0.001), while only DBP z-scores were related to cr-PWV (*p* < 0.01). BMI was neither associated with cf-PWV nor with cr-PWV values but was a strong predictor of LVMI (<0.001), whereas cardiac mass and BP z-scores were not related. Conclusions: Our study suggests that, in children, elevated BP values and excess weight may have different effects on the heart and the vessels in causing early cardiovascular alterations.

## 1. Introduction

The aorta and large elastic arteries play a fundamental role in the hemodynamics of the cardiovascular system. During the systolic phase of the cardiac cycle, thanks to their viscoelastic properties, the large arteries have an important buffer function on the left ventricular ejection. The potential energy stored in systole in the aortic wall is transformed into kinetic energy during the diastolic phase of the cardiac cycle (the Windkessel effect), performing an essential propulsive function [1]. Alterations in the viscoelastic properties of the aorta, due to stiffening of the arterial wall, can cause important changes in the systemic circulation. The first consequence is an increase in systolic blood pressure (SBP), with a consequent increase in cardiac work. This process is associated with a reduction in diastolic blood pressure (DBP), inducing a reduction in subendocardial perfusion and an increase in pulse pressure. Starting from these pathophysiological assumptions, several studies established arterial stiffness as an independent predictor of cardiovascular morbidity and mortality [2,3,4]. It is, therefore, very important to discover the presence of an early impairment of the distensibility of large elastic arteries by means of reliable non-invasive methods and to identify an increased cardiovascular risk at the pediatric age. The measurement of pulse wave velocity (PWV) represents a simple and reproducible method to evaluate the distensibility of an arterial segment in a non-invasive manner. The pulse waves propagate with a speed that is inversely related to the viscoelastic properties of the wall itself: the higher the PWV, the stiffer the arterial wall. At present, the measurement of carotid-femoral PWV (cf-PWV) is considered the non-invasive gold standard method to assess the aortic stiffness [2,5,6], and to evaluate the mechanical properties of large elastic arteries. The measurement of carotid-radial PWV (cr-PWV) provides, instead, useful information on the distensibility of the upper limb muscular arteries [1].

Several studies showed a positive association between cf-PWV and arterial BP values in children [7,8,9,10]. Mean arterial pressure, age and height are the major determinants of cf-PWV in childhood [2,11,12]. Higher values of cf-PWV were shown in pre-hypertensive and hypertensive children and adolescents compared to normotensives [10,13,14]. Moreover, a progressive increment in cf-PWV values from normotensive to hypertensive subjects has been reported in overweight and obese children and adolescents [15]. However, there is no agreement regarding the relationship between childhood excess weight and aortic stiffness. In fact, while some studies showed a positive association between elevated cf-PWV and excess weight [16,17,18], other studies, on the contrary, described an inverse association between body mass index (BMI) and cf-PWV [13,19]. Furthermore, it should be stressed that all previous studies investigating the relationship between BP and cf-PWV values referred to the absolute BP values measured at the time of the cf-PWV measurement. However, according to the guidelines, the values of BP in the children should be indexed, taking into account gender, age and height (i.e., BP z-score).

The aim of this study is to evaluate the relationship between BP and BMI z-scores and arterial stiffness, evaluated by measurement of cf-PWV and cr-PWV, in children. Furthermore, we also evaluated the relationship between BMI z-scores/body weight class and BP z-scores/BP category with left ventricular mass index (LVMI), to assess if different cardiovascular risk factors have different effect on early organ damage in childhood.

## 2. Methods

A cohort of children and adolescents consecutively referred to the Pediatric Center for Cardiovascular Risk Prevention of the Istituto Auxologico Italiano in Milan, from October 2013 to December 2018 was recruited. Exclusion criteria were age less than 5 or more than 18 years, secondary forms of hypertension, congenital cardiovascular diseases, history of aortic surgery, arrhythmias, impaired glucose tolerance, diabetes and renal insufficiency. Patients underwent a clinical evaluation, transthoracic echocardiography and arterial tonometry on the same day. Anthropometric parameters and clinical history were collected during the clinic visit. The study protocol was approved by the local institutional ethics committee and conformed to the ethical guidelines of the 1975 Declaration of Helsinki (RICARPE study, 2015102002). Informed consent was obtained from parents or legal representatives before the enrollment in the study.

### 2.1. Clinical Parameters

BMI was calculated as weight/height (kg/m^2^), and weight class was defined according to the International Obesity Task Force classification, distinguishing among normal weight, overweight, and obese classes [20]. BMI z-scores were calculated using the Center for Disease and Control prevention charts (CDC) reference [21]. The waist circumference was measured at the level midway between the lowest rib margin and the iliac crest. Waist-to-height ratio was calculated as waist circumference divided by height. BP was measured using an oscillometric device validated in children (Omron 705IT; Omron Co, Kyoto, Japan) with the appropriate cuff for the children’s upper-arm size. BP measurements were performed after at least 5 min of rest, with the child in a sitting position. BP was measured 3 times (at 3-min intervals) and the average of the last two measurements was considered [22] to classify the children as follows: normotensive if both SBP and DBP were <90th percentile; high normal if SBP and/or DBP values were between the 90th and 95th percentile; hypertensive if SBP and/or DBP were ≥95th percentile.

### 2.2. Echocardiography

Two-dimensional M-mode, pulsed-wave Doppler and tissue Doppler echocardiography analyses were obtained in the standard precordial positions using digital echocardiography equipment (Aloka ProSound SSD Alpha 10, Tokyo, Japan), with 1–5 MHz transducers and following the recommendations for standard M-mode measurements. Instantaneous measurements were made over three cardiac cycles and the average values were obtained from each patient for the following parameters: left atrial end-diastolic diameter (LAd), interventricular septum thickness at end diastole (IVSd), left ventricular posterior wall thickness at end diastole (LVPWd) and left ventricular end-diastolic diameter (LVEDd). Left ventricular mass (LVM) was calculated according to the American Society of Echocardiography convention and indexed (LVMI) to height (m^2.7^) [23]. Left ventricular hypertrophy was defined as the presence of a LVMI greater or equal than the 95th percentile specific for age and sex, according to the reference values by Khoury et al. [24].

### 2.3. Measurement of Arterial Stiffness

Measurements were obtained after 10 min of rest and at a stable room temperature. A validated, easy-to-use and high-fidelity PulsePen tonometer (DiaTecne srl, San Donato Milanese, Italy) was used in this study [25,26]. Reproducibility of the PulsePen device [27] and the procedure has been described in detail previously [25,28]. Briefly, the PulsePen consists of a pocket size, high-fidelity applanation tonometer and an integrated ECG unit. PWV was measured by recording carotid and peripheral (femoral or radial) waveforms in rapid succession. PWV was defined as the distance between the measuring sites divided by the time delay between the distal pulse wave from the proximal pulse wave, using the ECG trace as reference. As recommended from an expert consensus document on the measurement of aortic stiffness, 80% of the direct distance from the 2 sites was used to calculate the PWV [29]. The PulsePen device software did not validate measurements if the difference between BP or heart rate values taken at the time of carotid and peripheral (femoral or radial) artery recordings was >10%. The use of the PulsePen device in children was validated in a previous study, which provided reference values for cf-PWV in children and adolescents [7].

### 2.4. Statistical Analysis

The sample was categorized according to weight class and BP category. The continuous variables were described by mean and standard deviation (SD) and the distributions were compared across groups by one-way analyses of variance with α = 0.05 significance level. Post hoc pairwise comparisons were obtained according to the Bonferroni correction significance level (α/3). The categorical variables were described by the proportion of subjects falling into each category. Proportions were compared across groups by the Chi-Square test with post hoc pairwise comparisons obtained according to the Bonferroni correction significance level (α/3). The univariate association between cf-PWV (cr-PWV) and SBP (mmHg and z-score values), DBP (mmHg and z-score values), mean BP (MBP) and heart rate were represented in scatterplots, where a 95% confidence interval on the Pearson correlation test and the p-value were displayed. Similarly, the associations between LVMI and SBP (mmHg and z-score values) and DBP (mmHg and z-score values) and BMI (kg/m^2^ and z-score values) were represented in scatterplots with Pearson correlation statistics. A multiple linear regression model was used to assess the impact of age, gender, heart rate, BMI and SBP (or DBP) on the cf-PWV and on the cr-PWV and the impact of age, gender, BMI and SBP (or DBP) on the LVMI. All the analyses were conducted with the package R [30]. 

## 3. Results

A total of 343 children (57.7% males, average age in years 11.7, SD = 2.9) were enrolled in the study. Table 1 and Table 2 show the anthropometric and clinical characteristics of the study population according to weight class and BP category, respectively.

About 21% of the subjects were normal weight, 34% were overweight and 45% were obese. Obese children were younger than overweight children (*p* < 0.02). SBP and DBP z-scores increased significantly as the weight class worsened (*p* < 0.001 in both cases). LVMI values were significantly greater in overweight and obese compared to normal weight children (*p* < 0.02 in both cases), while the highest prevalence of left ventricular hypertrophy was in obese children (*p* < 0.02). There were no differences in cf-PWV and cr-PWV values according to weight class (Table 1).

The prevalence of hypertension was about 17%, while 10% of children showed high normal BP values. Hypertensive children were older than children with normal and high normal BP values (*p* < 0.02), whereas BMI z-score and waist-to-height ratio increased from normotensive to children with more elevated BP values (*p* < 0.02). Cf-PWV values were significantly increased in higher BP categories compared to normotensive children (*p* < 0.02 in both cases). Cr-PWV values were significantly higher in high normal (BP) subjects compared to normotensives (*p* < 0.02). There were no differences in LVMI among the BP categories (Table 2).

**Figure 1:**
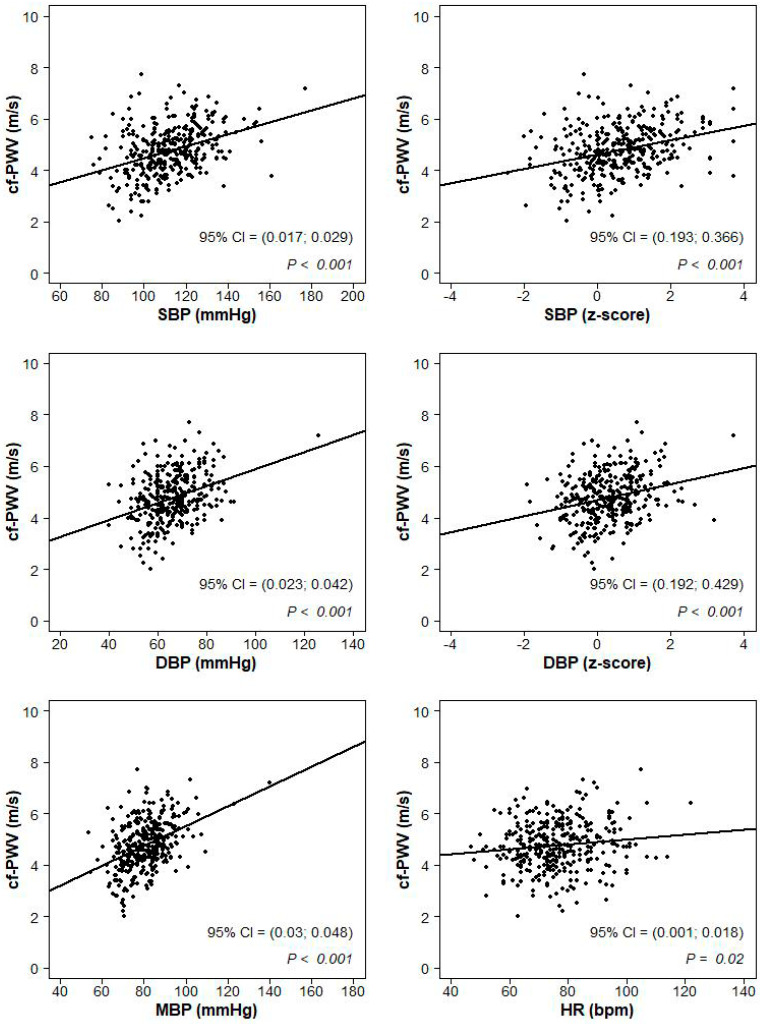
Scatterplots of the association between carotid femoral-pulse wave velocity (cf-PWV) and systolic blood pressure (SBP; mmHg and z-score values), diastolic blood pressure (DBP; mmHg and z-score values), mean blood pressure (MBP; mmHg) and heart rate (HR; beats per minute).

**Figure 2:**
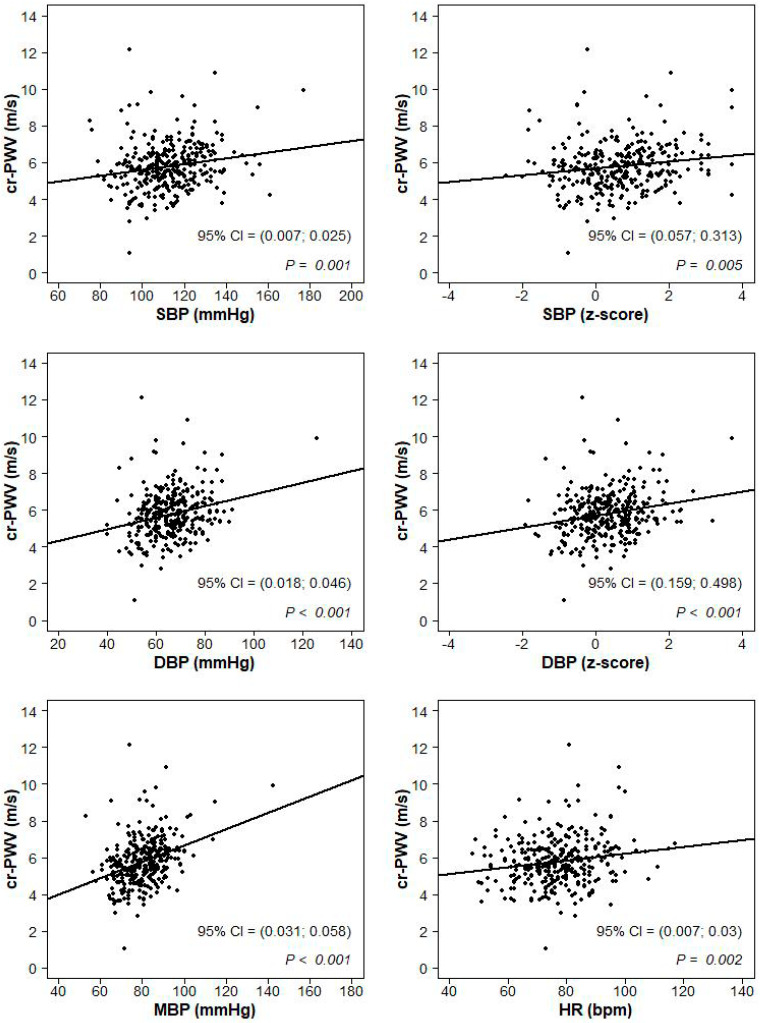
Scatterplots of the association between carotid radial-pulse wave velocity (cr-PWV) and systolic blood pressure (SBP; mmHg and z-score values), diastolic blood pressure (DBP; mmHg and z-score values), mean blood pressure (MBP; mmHg) and heart rate (HR; beats per minute).

At univariate analysis, cf-PWV (Figure 1) and cr-PWV (Figure 2) values were correlated to SBP, DBP, mean BP, SBP z-scores, DBP z-scores and heart rate (*p* < 0.02).

Multiple linear regression models showed that age, heart rate, SBP and DBP z-scores were significantly associated with cf-PWV (*p* < 0.01, Table 3), while age, gender, heart rate and DBP z-scores were related to cr-PWV (*p* < 0.05, Table 4). BMI was neither associated with the cf-PWV values nor with those of cr-PWV.

**Figure 3:**
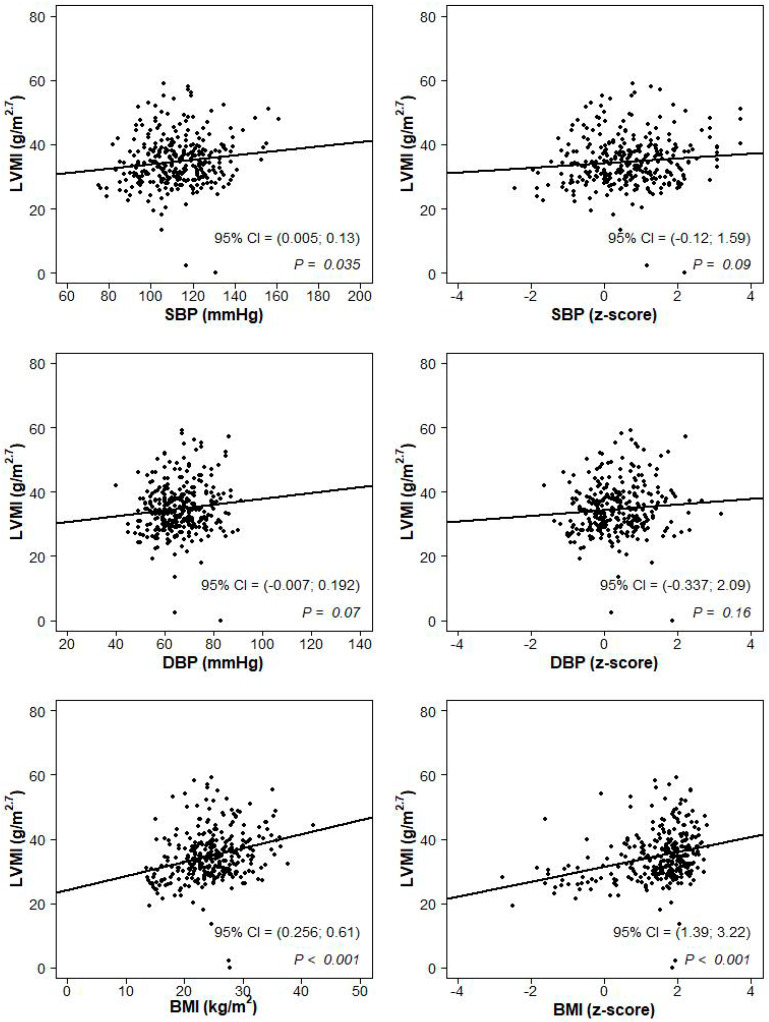
Scatterplots of the association between left ventricular mass index (LVMI; g/m^2.7^) and systolic blood pressure (SBP; mmHg and z-score values), diastolic blood pressure (DBP; mmHg and z-score values) and body mass index (kg/m^2^ and z-score values).

At univariate analysis, LVMI was strongly correlated with BMI (*p* < 0.001 for both kg/m^2^ and z-scores values, Figure 3). There was also a correlation, albeit somewhat weaker, between LVMI and SBP (*p* = 0.035, Figure 3).

From the multiple linear regression model in Table 5 it can be noted that only gender and BMI z-scores were significantly associated with LVMI (*p* = 0.026 and *p* < 0.001, respectively), while the relationship between cardiac mass and SBP z-scores disappeared.

When body weight and BP were included as categorical variables (weight class and BP category), multiple regression analysis showed that the presence of hypertension was strongly associated with increased values of cf-PWV (*p* = 0.008), while overweight and obesity were associated with increased values of LVMI (*p* = 0.028 and *p* < 0.001, respectively). Heart rate was shown to be significantly associated with both cf-PWV and cr-PWV (*p* < 0.001 and *p* = 0.001, respectively). If body weight was included in the multivariate models as central adiposity (waist-to-height expressed as a continuous variable or as waist-to-height ratio >50%) results did not change significantly.

## 4. Discussions

Our study provides several new results; (i) indexed values of SBP and DBP, i.e., z-scores, are strongly associated with cf-PWV values in a pediatric population; (ii) SBP z-score is not a predictor of cr-PWV values, while heart rate and DBP z-score are significantly related with cr-PWV; (iii) BMI z-score is a strong predictor of LVMI, while there is no relationship between BMI z-score and cf-PWV and cr-PWV values.

Our study demonstrates a relationship between the indexed values of SBP and DBP (i.e., SBP and DBP z-scores) and PWV. Several studies showed higher PWV values in hypertensive than normotensive children and adolescents [7,8,9,10]. Our findings confirm the association between hypertension and PWV, but take a further step in understanding the relationship between arterial pressure and vascular stiffness, demonstrating that, during childhood and adolescence, when the cardiovascular system is developing, an increase in SBP and DBP z-scores leads to an increase in PWV, regardless of the presence of a diagnosis of arterial hypertension. Pediatricians recommend not to use absolute values of anthropometric and hemodynamic variables in children and adolescents, but to index them according to gender, age and height of the subject. An examination like the measurement of PWV, may arouse a certain apprehension and emotion in a child, leading to a reaction similar to the so-called “white coat effect”, with an increase of both BP and heart rate. The results of our study suggest that BP values assessed as indicated by the pediatric guidelines, i.e., BP z-score calculated on different measurements obtained in resting conditions [21], could be a more important predictor of a child’s arterial stiffness than the office BP values at the time of the examination. Not many studies are available in which the authors adjusted PWV data for all possible confounding factors (i.e., indexed values of BP and BMI/central adiposity, age, gender, puberty and heart rate), and, to our knowledge, no study at all adjusted the multivariate analysis models for BP z-scores, instead of simply adjusting for BP raw values, and also for heart rate, in the evaluation of PWV in healthy children. Indeed, a several studies showed, both in animals and humans, an inverse relationship between heart rate and vascular distensibility [31,32,33].

In our population, a relationship between SBP z-scores and PWV was present only for cf-PWV, and not for cr-PWV, which in turn was significantly associated with DBP z-scores and heart rate values only. While cf-PWV is a reliable index of viscoelastic properties of the aorta and large elastic arteries, cr-PWV assesses the mechanical properties of the muscular upper limb arteries. Thus, while the cf-PWV increases above all in the presence of structural alterations of the aortic wall, the aorta being poorly innervated by the sympathetic system, on the other hand, cr-PWV reflects the functional condition of the arterial tree, which is closely related to the activation of the sympathetic system. This explains the close link between cr-PWV and diastolic BP and heart rate. However, it does not mean that the sympathetic system cannot influence the distensibility of the aorta; indeed, the sympathetic nervous system promotes arterial stiffness independent of blood pressure via endothelial dysfunction, growth of vascular muscle and associated fibrosis, by its effects on the renin-angiotensin aldosterone system, promoting arterial wall fibrosis. This influence of the sympathetic system on arterial distensibility occurs both on the aorta as well as on muscular arteries. Moreover, while cf-PWV increases significantly with aging, cr-PWV does not change with age. At present, no study has shown any prognostic or clinical significance of high cr-PWV values.

The results of our study suggest the absence of any association between BMI and PWV, after correction for possible confounding factors. Other studies report contradictory results on the relationship between BMI and arterial stiffness. Lurbe et al. [13], Hvidt et al. [19] and Pierce et al. [18] showed lower cf-PWV values in populations of obese children and adolescents when compared with the non-obese (*n* = 501, *n* = 141 and *n* = 227, respectively). On the contrary, Urbina et al. showed an increased arterial stiffness in 195 diabetic adolescents and young adults with obesity compared to 241 lean controls and to 234 non-diabetic obese subjects [16]. In a pilot study, involving a small sample of normal weight (*n* = 27) and obese children (*n* = 22), Koopman et al. found a positive association between BMI and cf-PWV values, after adjusting for SBP and heart rate [17]. More recently, Dangardt et al. [34] showed, in a large population of children and adolescents (*n* = 3423), that persistence of high total fat mass during adolescence, assessed using dual-energy x-ray absorptiometry (DEXA), was positively associated with increased cf-PWV. This adverse association was absent with BMI or waist-to-height ratio [34]. The absence of an association in our study between central obesity and PWV could be due to the fact that visceral fat was not quantified by DEXA.

A completely new finding of our study is that high BP values and excess weight exert a different effect on arterial distensibility and on cardiac mass, causing early differentiated damage to target organs. In a previous study, performed in a larger number of subjects (*n* = 526), we demonstrated that BP z-score and BMI z-score both play a role in influencing LVMI values. However, while BP retained its independent predictive value, the role of BMI in determining the increase of LVMI and of the presence of left ventricular hypertrophy was much greater [35]. Moreover, obesity but not hypertension, was associated with a worsening of diastolic function, and excess weight and hypertension showed different effects on left ventricular geometry. In the present study, in which the prevalence of subjects with excess weight is very high, the BP z-score fails to maintain the role of an independent predictor of LVMI, but remains highly significant in determining PWV values. We can, therefore, hypothesize that weight and BP exert two independent and quantitatively different effects on the large arteries and on the heart in children. Therefore, our study strongly emphasizes that not only children showing both hypertension and obesity should be carefully followed-up, but also those that present one of the two clinical conditions only. In particular, it may be hypothesized that early vascular damage occurs first in hypertensive children and that, on the other hand, cardiac damage precedes vascular alterations in childhood obesity.

The main limitation of the study is the observational approach that does not allow us to assess the causal association between BP values and aortic distensibility, evaluated by measurement of the cf-PWV. These two parameters likely influence each other, and more studies are needed to understand how aortic stiffening affects BP and how an increase in BP affects PWV in children. Another limitation of the study is that total fat mass was not measured. In our research, only BMI, waist circumference and waist-to-height ratio were considered. In a similar population, Dangardt’s study [32] showed a positive association between total fat mass and arterial stiffness and a lack of association between BMI and aortic PWV. Interpreting these apparently conflicting results, we must consider that the association between BMI and body fat is weak in childhood but becomes strong in adulthood. Our research supports the hypothesis that, in a pediatric population, a more detailed phenotype is probably needed to assess the relationship between adiposity and vascular damage.

## 5. Conclusions

In conclusion, this study showed a strong association between BP z-scores and cf-PWV values, and a significant association between BMI z-score and LVMI. No association was found between the BMI z-score and cf-PWV values. Our study suggests that already in the pediatric age there is a close relationship between high blood pressure and vascular stiffness and that the impact of high blood pressure and weight excess is different if we consider two different indices of early organ damage. Only prospective studies will be able to demonstrate whether the described relationship of hypertension and weight excess with early organ damage in children may constitute a real cardiovascular risk factor in the future adult as well.

## Figures and Tables

**Table 1:** Anthropometric and clinical characteristics according to weight class.

Parameter	NW (*n* = 71; 20.7%)	OW (*n* = 116; 33.8%)	OB (*n* = 156; 45.5%)	*p*
Age (years), mean (SD)	11.5 (3.3)	12.3 (2.5)	11.1 (2.8) #	0.003
Gender (males), *n* (%)	41 (57.7)	71 (61.2)	86 (55.1)	0.60
Systolic BP (mmHg), mean (SD)	107.2 (18.6)	113.0 (13.3)	114.4 (14.1) *	0.003
Diastolic BP (mmHg), mean (SD)	62.2 (12.6)	64.6 (8.8)	68.2 (8.7) *#	<0.001
Systolic BP (z-score), mean (SD)	0.19 (1.20)	0.57 (0.98)	0.83 (1.06) *	<0.001
Diastolic BP (z-score), mean (SD)	0.01 (0.91)	0.17 (0.74)	0.54 (0.75) *#	<0.001
BP category, *n* (%)				0.042
normotensive	61 (85.9)	88 (75.9)	104 (66.7)	
high normal	3 (4.2)	11 (9.5)	19 (12.2)	
hypertensive	7 (9.9)	17 (14.7)	33 (21.2)	
cf-PWV (m/s), mean (SD)	4.7 (1.1)	4.8 (0.9)	4.8 (0.8)	0.48
cr-PWV (m/s), mean (SD)	5.7 (1.4)	6.0 (1.3)	5.7 (1.2)	0.14
LVMI (g/m^2.7^), mean (SD)	31.8 (7.3)	34.8 (8.7) *	36.1 (8.2) *	0.003
LVH, *n* (%)	8 (12.9)	20 (19.4)	45 (30.0) *	0.015

SD, standard deviation. BP, blood pressure; cf-PWV, carotid-femoral pulse wave velocity; cr-PWV, carotid-radial pulse wave velocity; LVMI, left ventricular mass index; LVH, left ventricular hypertrophy; NW, normal weight; OW, overweight, OB, obese. Post hoc pairwise comparisons *p*-values < α/3: *, versus NW; #, OB versus OW.

**Table 2:** Anthropometric and clinical characteristics according to blood pressure category.

Parameter	NT (*n* = 253; 73.8%)	HN (*n* = 33; 9.6%)	HT (*n* = 57; 16.6%)	*p*
Age (years), mean (SD)	11.1 (2.8)	12.2 (2.9)	13.4 (2.4) *	<0.001
Gender (males), *n* (%)	146 (57.7)	20 (60.6)	32 (56.1)	0.92
Waist (cm), mean (SD)	75.9 (12.9)	84.2 (12.6) *	86.5 (12.2) *	<0.001
BMI (kg/m^2^), mean (SD)	23.5 (4.7)	26.7 (4.6) *	27.5 (4.8) *	<0.001
BMI (z-score), mean (SD)	1.36 (1.03)	1.77 (0.75) *	1.69 (0.67) *	0.010
WtHr (%), mean (SD)	51.8 (6.9)	55.7 (7.4) *	54.0 (7.7)	0.004
WtHr > 50%, *n* (%)	160 (63.7)	24 (75.0)	39 (70.9)	0.31
Weight class, *n* (%)				0.042
normal weight	61 (24.1)	3 (9.1)	7 (12.3)	
overweight	88 (34.8)	11 (33.3)	17 (29.8)	
obese	104 (41.1)	19 (57.6)	33 (57.9)	
cf-PWV (m/s), mean (SD)	4.6 (1.0)	5.1 (0.8) *	5.2 (0.8) *	<0.001
cr-PWV (m/s), mean (SD)	5.7 (1.3)	6.3 (1.1) *	6.1 (1.2)	0.013
LVMI (g/m^2.7^), mean (SD)	34.6 (8.3)	34.6 (7.7)	35.6 (8.9)	0.73
LVH, *n* (%)	49 (21.4)	7 (21.9)	17 (31.5)	0.28

SD, standard deviation. BMI, body mass index; WtHr, waist-to-height-ratio; cf-PWV, carotid-femoral pulse wave velocity; cr-PWV, carotid-radial pulse wave velocity; LVMI, left ventricular mass index; LVH, left ventricular hypertrophy; NT, normotensive; HN high-normal blood pressure values; HT, hypertensive. Post hoc pairwise comparisons *p*-values < α/3: *, versus NT.

**Table 3:** Effect of age, gender, heart rate, BMI and systolic BP (Model A) or diastolic BP (Model B) on carotid-femoral pulse wave velocity (m/s) by a multiple linear regression model.

Variable	Model A			Model B		
	b	(95% CI)	*p*	b	(95% CI)	*p*
Intercept	−0.097	(−0.305; 0.111)	0.36	−0.075	(−0.281; 0.132)	0.48
Age (years)	0.295	(0.180; 0.411)	<0.001	0.347	(0.241; 0.454)	<0.001
Systolic BP (z-score)	0.191	(0.089; 0.293)	<0.001		-	
Diastolic BP (z-score)		-		0.248	(0.120; 0.376)	<0.001
Heart rate (beats/min)	0.205	(0.096; 0.314)	<0.001	0.214	(0.106; 0.321)	<0.001
Gender (males)	−0.050	(−0.255; 0.156)	0.63	−0.023	(−0.229; 0.182)	0.82
BMI (z-score)	0.007	(−0.100; 0.113)	0.90	0.009	(−0.097; 0.114)	0.87

b indicates multivariate standardized coefficient; CI, confidence interval; BMI, body mass index; BP, blood pressure.

**Table 4:** Effect of age, gender, heart rate, BMI and systolic blood pressure (Model A) or diastolic blood pressure (Model B) on carotid-radial pulse wave velocity (m/s) by a multiple linear regression model.

Variable	Model A			Model B		
	b	(95% CI)	*p*	b	(95% CI)	*p*
Intercept	0.170	(−0.052; 0.393)	0.13	0.168	(−0.052; 0.387)	0.13
Age (years)	0.279	(0.151; 0.407)	<0.001	0.272	(0.156; 0.388)	<0.001
Heart rate (beats/min)	0.227	(0.105; 0.350)	<0.001	0.211	(0.093; 0.330)	0.001
Gender (males)	−0.257	(−0.482; −0.031)	0.026	−0.225	(−0.449; 0.000)	0.05
Diastolic BP (z-score)		-		0.198	(0.062; 0.334)	0.004
Systolic BP (z-score)	0.052	(−0.059; 0.164)	0.35		-	
BMI (z-score)	−0.035	(−0.059; 0.164)	0.55	−0.066	(−0.177; 0.044)	0.24

b indicates multivariate standardized coefficient; CI, confidence interval; BMI, body mass index; BP, blood pressure.

**Table 5:** Effect of age, gender, BMI and systolic blood pressure (Model A) or diastolic blood pressure (Model B) on left ventricular mass index (g/m^2.7^) by a multiple linear regression model.

Variable	Model A			Model B		
	b	(95% CI)	*p*	b	(95% CI)	*p*
Intercept	28.199	(23.941; 32.457)	<0.001	28.228	(24.174; 32.283)	<0.001
Age (years)	0.180	(−0.169; 0.528)	0.31	0.176	(−0.147; 0.499)	0.28
Gender (males)	2.059	(0.252; 3.867)	0.026	2.069	(0.255; 3.883)	0.025
BMI (z-score)	2.303	(1.340; 3.266)	<0.001	2.289	(1.334; 3.244)	<0.001
Systolic BP (z-score)	−0.001	(−0.941; 0.938)	0.99		-	
Diastolic BP (z-score)		-		0.058	(−1.183; 1.298)	0.93

b indicates multivariate coefficient; CI, confidence interval; BMI, body mass index; BP, blood pressure.
